# How schools can aid children’s resilience in disaster settings: The contribution of place attachment, sense of place and social representations theories

**DOI:** 10.3389/fpsyg.2022.1004022

**Published:** 2022-09-12

**Authors:** Emily-Marie Pacheco, Elinor Parrott, Rina Suryani Oktari, Helene Joffe

**Affiliations:** ^1^Clinical, Educational and Health Psychology, UCL Division of Psychology and Language Sciences & EPICentre, University College London, London, United Kingdom; ^2^Tsunami and Disaster Mitigation Research Centre, Department of Family Medicine, Faculty of Medicine, Universitas Syiah Kuala, Banda Aceh, Indonesia

**Keywords:** resilience, children, schools, disaster, place attachment, social representations, sense of place, risk management

## Abstract

Disasters incurred by natural hazards affect young people most. Schools play a vital role in safeguarding the wellbeing of their pupils. Consideration of schools’ psychosocial influence on children may be vital to resilience-building efforts in disaster-vulnerable settings. This paper presents an evidence-based conceptualization of how schools are psychosocially meaningful for children and youth in disaster settings. Drawing on Social Representations and Place Attachment Theories, we explore the nature of group-based meaning-making practices and the meanings that emerge concerning school environments in disaster settings. We contribute a novel understanding of how schools may mitigate psychosocial risk for young people by considering how schools are conceptualised at four levels: (1) as physical environment, (2) as social arena, (3) as a place with individual and (4) group-based significance. In each of these domains schools can foster disaster resilience in young people. This paper highlights the evidence concerning the functions of schools beyond their capacity as educational institutions, critically considering their social and physical functions in their communities. This evidence can inform stakeholders involved in disaster resilience building.

## Introduction

Children are uniquely vulnerable to the negative consequences of disasters, in part due to their dependence on adults and their ongoing development (Peek, 2008). Schools are essential sites for safeguarding children and youth in disaster settings (Mutch, 2014; see also, UNISDR, 2014; ACFCSS, 2016; Paci-Green et al., 2020). Though the significance of their role as educational institutions and resource distribution centers in disaster contexts is well-established (e.g., Sakurai et al., 2018; Mirzaei et al., 2019), consideration of schools’ psychosocial influence may be vital to disaster risk management and resilience-building (IASC, 2006; Pacheco et al., 2021). Many works explore the socio-physical dynamics of spaces such as cities or the home (e.g., Bechtel, 2010; Clayton, 2012; Fleury-Bahi et al., 2017; Sawyer et al., 2022), yet to our knowledge, the literature lacks academic conceptualization of the socio-physical elements of schools. This conceptual analysis explores the evidence regarding how children conceptualize schools in disaster settings; it examines how schools may mitigate psychosocial risk for children by considering the function of these conceptualizations from a social psychological perspective. We conclude with a series of recommendations for utilizing schools as hubs for enhancing resilience in disaster contexts.

Before we begin our conceptual analysis, we briefly clarify definitions of key concepts used throughout this paper. Since the United Nations International Strategy for Disaster Reduction adopted the Hyogo Framework for Action (HFA) 2005–2015: ‘*Building the Resilience of Nations and Communities’*, disaster policy, planning and research has focused on the capacity of the community to bounce back from adversity. Yet despite the rise in popularity of the resilience rhetoric, there has been a lack of interdisciplinary consensus on how the concept is defined (Mayunga, 2007; Bonanno et al., 2015). We view it as the ‘adaptive capacity’ that supports individuals and the community to cope with and recover from adversity (e.g., Berkes, 2007; Paton, 2007; Norris et al., 2008; Masten, 2011; Ungar, 2011). The adversity we refer to is that experienced in settings affected by disasters. While disasters vary in the scale of disruption and loss of life caused, a disaster setting is an area that has experienced widespread human, material, economic and/or environmental loss as a result of the interaction of a hazard (e.g., an earthquake, tsunami, flooding) with social vulnerability (Massazza et al., 2019). Resilience building is facilitated through a system of processes that buffer the impact of such disaster scenarios and improve circumstances in both the short-term response and long-term planning (Pacheco et al., 2021).

## Background and framework

This section explores how spaces can protect the psychosocial resilience of individuals and communities; we approach this through lenses of social representations theory (SRT), sense of place and place attachment theory. Subsequent sections apply this knowledge to understand the significance of schools (as physical and social environments) for children and youth in disaster settings. Within this paper, ‘schools’ refers to primary and secondary, but not tertiary, education.

### Social representations theory

Social representations are the product of group-based meaning-making practices whereby groups socially construct common knowledge on topics of social relevance (Moscovici, 1961/1976, 1984; Clémence, 2001). Abstract concepts are made concrete by their transformation into elements that are easier for people to engage with and discuss, such as integrating the concept into images or examples with relevance to everyday life (Clémence, 2001). The objectified concepts become fully integrated into contemporary meaning systems when connected to pre-existing meaning systems (Joffe, 2003). Such representations exist not only in belief and discourse but influence, and are inseparable from, social behavior (Sammut and Howarth, 2014; Wagner, 2015). It is therefore useful to draw on SRT to explore the symbolic meaning with which schools are infused in disaster settings.

People can differ in how they represent an entity. When people represent important social issues, their pre-existing cognitive-emotional frameworks are imposed upon the newer ideas; groups within the wider public draw on diverse information sources to understand a phenomenon (Abreu Lopes and Gaskell, 2015). The pre-existing frameworks are shaped by the complex social worlds within which the people exist (e.g., religious, cultural, ethnic, political, socioeconomic and ideological; Clémence, 2001; Staerklé et al., 2011; Wagner, 2012) so that contemporary societies experience a plurality of representations of the same object (Abreu Lopes and Gaskell, 2015). Such frameworks influence belief structures, life experience and knowledge acquisition (Staerklé et al., 2011); they become more salient when a threat is encountered (Joffe, 2003; Jaspal et al., 2022).

Although SRT has been applied to understanding risk (Joffe, 2003; Lemée et al., 2019) and representations of the home (Harries, 2013) in disaster settings, it has yet to be applied to understanding the content or implications of conceptualizations concerning schools in disaster settings.

### Sense of place

The literature provides a plethora of definitions and concepts for characterizing the complex processes whereby humans develop connections to places, such as place attachment (Hidalgo and Hernández, 2001; Altman and Low, 2012; Scannell and Gifford, 2016, 2017; Manzo and Devine-Wright, 2020), sense of community (McMillan, 1996; Obst et al., 2002), sense of place (Jorgensen and Stedman, 2001; Pretty et al., 2003; Silver and Grek-Martin, 2015), place identity (Twigger-Ross and Uzzell, 1996; Devine-Wright, 2009; Foroudi et al., 2020), rootedness (Relph, 1976; Tuan, 1980), belonging (Inalhan and Finch, 2004; Rogaly and Taylor, 2016; Di Masso et al., 2017), place-making (Trudeau, 2006; Friedmann, 2010; Pierce et al., 2011; Ujang, 2012) and making sense of place (Matthews, 1992; Relph, 2009; Powell, 2010; Convery et al., 2014). Each of these terms appears across disciplines interested in place-related research (e.g., urban studies, psychology, human geography, sociology), and has been operationalized across the research literature within concrete variables (e.g., quantitatively captured in years lived in an area) as well as abstract variables (e.g., qualitatively captured in how one understands one’s experience of or in a place; see Lewicka, 2010; Williams, 2014; Greer et al., 2020). While many authors use these terms interchangeably, a rich literature is dedicated to untangling each of these concepts (e.g., Hashemnezhad et al., 2013; Collins-Kreiner, 2020). Lewicka (2010), who provides an extensive review of several hundred empirical and theoretical works, argues that the literature should turn away from pursuit of defining these terms within rigid parameters and, instead, work toward extending theory and conceptualizations of person-place attachments in under-researched populations and settings. We respond to this challenge: there is little previous thought concerning the meaning ascribed to schools within communities, and none, to our knowledge, concerning this topic in disaster settings.

Schools’ meaning in disaster settings can be informed by understanding how individuals and groups develop psychological ties to places. All environments are social and physical (socio-physical), allowing person-place interactions to be bidirectional on a series of interacting levels: The thoughts, feelings, and behaviors of a person influence the elements of a place, and the elements of a place influence the thoughts, feelings, and behaviors of the individual experiencing that place (Sörqvist, 2016). Places contain three components: location (i.e., absolute and relative space), locale (i.e., material features that exist in that space), and sense of place (i.e., affective interactions with elements of that space; Silver and Grek-Martin, 2015). Location and locale refer to the external elements (built and natural) of a space. Sense of place refers to the affective psychological orientation – memories and experiences - that individuals or groups have in relation to a spatial setting. These contribute to the location and locale becoming a meaningful place (Jorgensen and Stedman, 2001; Silver and Grek-Martin, 2015). Jorgensen and Stedman (2006) further describe sense of place as a general complex psychosocial structure that organizes beliefs, emotions, and behaviors. Since sense of place acknowledges that the meaning people ascribe to a place is a dynamic, multidimensional product of subjective processes, disaster studies tend to favor sense of place as a lens to explore the psychosocial ways in which communities have been affected by natural hazards (e.g., Chamlee-Wright and Storr, 2009; Smith and Cartlidge, 2011; Silver and Grek-Martin, 2015; Bonaiuto et al., 2016). Thus, sense of place research strongly informs and guides this work in conceptualizing schools’ symbolic and affective meaning for communities in disaster settings.

### Place attachment

Most scholars consistently uphold that sense of place is a product of place attachment (e.g., Shamsuddin and Ujang, 2008; Kudryavtsev et al., 2012). Even though the literature lacks a clear, single definition of place attachment, it is most consistently described as the subjective bonds people develop with particular places they hold important or meaningful (Hidalgo and Hernández, 2001; Greer et al., 2020). Place attachment has commonality with attachment to a person (Fullilove, 1996), encompassing the emotional and cognitive experience linking people to places (Bonaiuto et al., 2016) and captures the meaning individuals make of their environments and how they interact with those environments (Lewicka, 2005; Greer et al., 2020, p. 307–308).

Scannell and Gifford (2010) synthesize the literature concerning the various elements that contribute to place attachment in a well-cited and evidence-based tripartite model that envisions place attachment as a product of three dynamic elements. The *person* element concerns the actor who is attached; *place* concerns the object of attachment, including concrete and abstract elements of a place to which one is attached; psychological *process* refers to (how the attachment manifests; psychological elements of attachment; see also, Lewicka, 2011; Counted, 2016; Manzo and Devine-Wright, 2020). To contextualize this framework for disaster settings, Jamali and Nejat (2016) provide a broad conceptual map of demographic, socioeconomic, spatial and psychological factors that act as ‘parameters of place attachment’. However, they do not delve into the psychological factors. In a review of the literature on community disaster risk reduction, recovery, and resilience as well as place attachment and young people’s experiences of place in disaster settings, Scannell et al. (2016) argue that place attachment is important for young people’s experiences at each stage of the disaster (e.g., preparedness, experience, recovery and resilience). However, they find that while themes relevant to place attachment are often mentioned in empirical findings in disaster social science research, they are rarely discussed in the context of specific place attachment theory, especially in research on children and youth. A social psychological lens has been notably missing from the place attachment and disaster resilience discourses (Lewicka, 2011; Pacheco et al., 2021). This paper endeavors to address this gap as a social psychological lens can help us to conceptualize how places (i.e., schools) can act as icons of recovery for youth (Cox et al., 2017), such as by fostering emotional regulation and positive affect.

## Framework and method

This paper seeks to explore how pupils represent schools in disaster settings and the implications of these representations for resilience building. Existing evidence is assimilated and combined to support arguments using a theory synthesis (Jaakkola, 2020). This is a conceptual integration across multiple literature streams. It offers an enhanced view of a phenomenon by linking previously unconnected pieces in a novel way.

**Figure 1:**
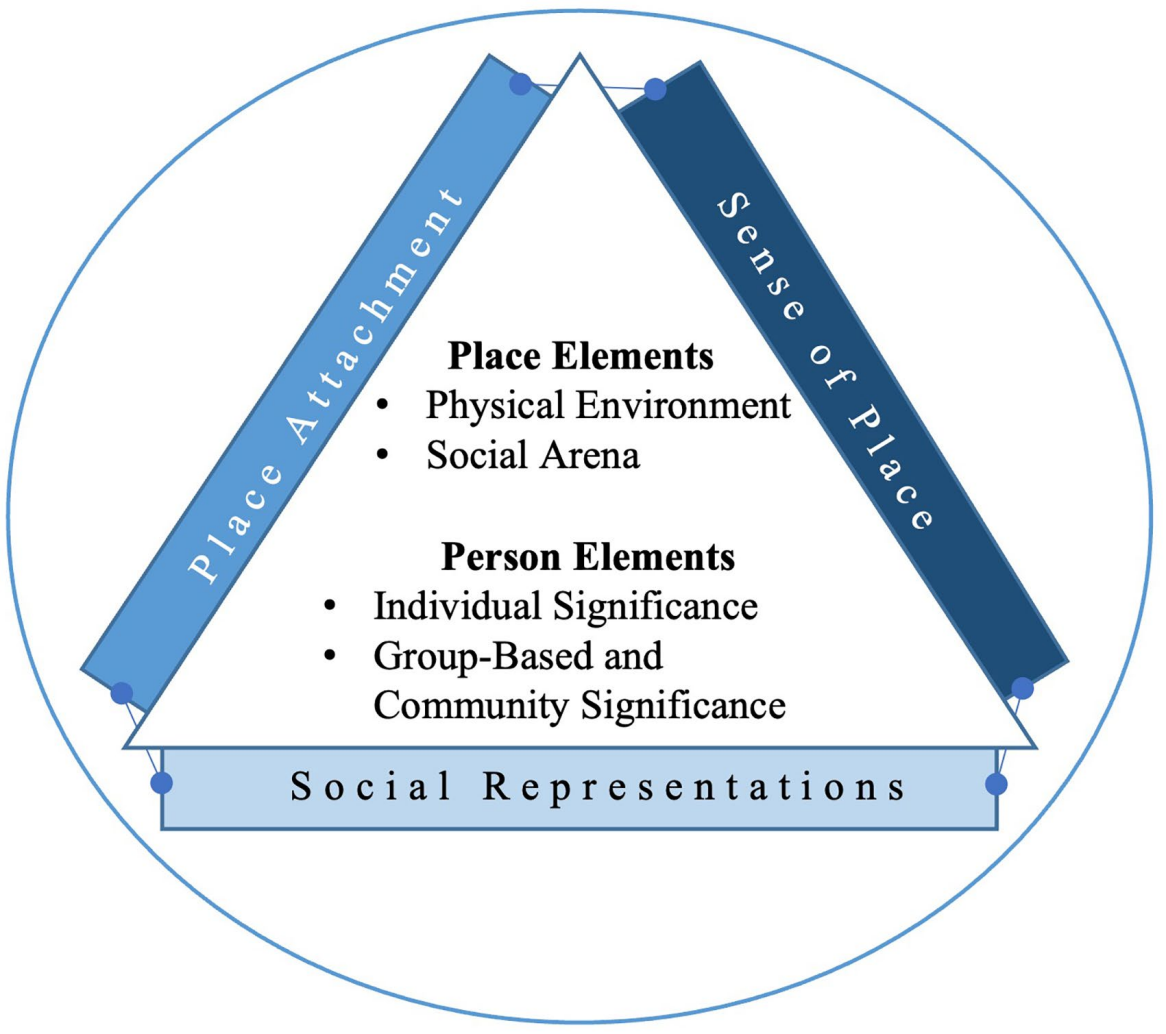
Conceptual framework for understanding the functional resilience building elements of schools. We consider how schools may mitigate psychosocial risk for young people in disaster settings across four elements; these elements are conceptualised based on a unified framework (lens) between place attachment, sense of place and social representations theories.

This paper seeks to synthesize the sense of place, place meaning and place attachment literatures insofar as they characterize the person-place bond (see Figure 1). This bond contains psychosocial, affective and bidirectional (see also, Lewicka, 2011) facets. Doing so provides an academic foundation for understanding how schools are ascribed meaning by people in disaster contexts (sense of place) and the socio-physical elements of schools that may influence these representations (place meaning and place attachment). We also consider the nature of individual and group-based (i.e., community) ties to schools in disaster settings and explore identity dynamics that contribute to these environments being represented as personally and symbolically significant.

## Schools as meaningful places in disaster settings

Young people’s representations of schools are characterized by cognitive-affective meaning. Beyond their capacity as educational institutions, schools provide their communities with necessary, supportive resources during disaster response and recovery phases. They are often repurposed as shelters or evacuation centers (Mutch, 2015). Beyond these practical functions, schools are also important social environments to which people develop physical, moral, social, emotional, spiritual, aesthetic, and academic attachment (Noddings, 2005; Rich and Schachter, 2012). Such attachments are especially salient for young people and may endure over the life course. For example, young people often represent schools as protective spaces (Sinkkonen, 2012). Research suggests young adults continue to retain these representations; Scannell and Gifford (2017) found that spontaneously visualizing familiar places, including schools, enhanced undergraduates’ sense of belonging, self-esteem, and meaningfulness. These findings suggest that schools have enduring socio-physical qualities and demonstrate that the person-place bond between pupils and schools may provide psychological benefits.

We structure our conceptualization of the place meaning of schools for children and youth in disaster settings by considering the meaning of schools as (1) physical built environments, (2) social arenas, (3) places with personal, and (4) group-based significance. We draw on the Scannell and Gifford (2010) model of place attachment as it provides a systematic pathway through the core elements that interact in fostering a place bond.

### Schools as physical places and built-environments

The concept of place, most commonly defined as space endowed with meaning (Relph, 1976; Tuan, 1977; Low and Altman, 1992), is the object of attachment within place-attachment theory (Scannell and Gifford, 2010). It is well-acknowledged that the physical attribute of ‘place’ has been under researched compared to the over-emphasis on the social dimension of place attachment (Stedman, 2003; Droseltis and Vignoles, 2010; Lewicka, 2011; Sebastien, 2020). This absence is particularly notable concerning young people in the disaster literature (Cox et al., 2017). In child samples much of the recent research concerning the meaning of physical places considers the importance of green spaces (e.g., see Little and Derr, 2020 for a review) and the promotion of pro-environmental behaviour through place attachment (Cole et al., 2021). In this body of research, the scale of meaningful built environments in a child’s life vary, from the small-scale (e.g., a bedroom) to the large scale (e.g., a city; Little and Derr, 2020). For children and youth, the physical aspects of such environments may be more salient than for adults. Morgan (2010) explains that adults’ attachment is driven by their feelings of a place and the meaning attributed to those feelings. In contrast, children understand places based on what one can do in the place (e.g., play, self-directed exploration) with little regard for the purpose of the place or the social meanings. As the environment is a passive element in relation to the activity, the bonds fostered in children are initially unconscious but become conscious as children are involved in repeated person-environment interactions and begin to develop feelings about those interactions (Jack, 2010). Significant physical places can benefit young people by satisfying physical and emotional needs, as they have been found to provide a sense of comfort that supports cognitive restoration and emotional regulation (Korpela et al., 2002). Important physical places can also provide a symbolic function. In disaster settings there is emerging evidence that a range of physical places, including the home and school, become symbols of recovery for young people. For example, arts workshops involving youth between the ages of 13–22 across four disaster-affected communities in the United States and Canada highlighted key people, places, and activities that supported their recovery (Cox et al., 2017); these insights were based on local knowledge and lived experience, demonstrating that it is important to collect and document youth perspectives when contextualizing theories of disaster recovery. The finding that physical places symbolize recovery after disaster has been well-evidenced in adult samples (Cox and Perry, 2011; Silver and Grek-Martin, 2015).

The development of person-place bonds varies according to developmental need (Morgan, 2010), and the role of schools in children’s lives continues to evolve, as do the nature of the interactions. For example, the journeys to and from school are important person-place interactions for children. They provide valuable opportunities for unstructured interaction with their social and physical worlds, which contribute to the development of their personal and community identities (Jack, 2010; see also O'Brien et al., 2000; Ross, 2007; Scannell and Gifford, 2017). These journeys allow children to actively engage with local space, contributing to secure attachments to the broader school locale and belonging to place (Jack, 2010). Future research could explore whether different modes of transport have unique psychological benefits, for example whether walking may foster attachment to the locale whereas a car journey may foster parent–child attachments. After Hurricane Katrina Forthergill and Peek (2015) conducted interviews and observations of children that revealed playgrounds and ball fields to be important places for recovery. Overall existing research supports the notion that schools exist as important physical places in children’s lives as they provide a context that scaffolds developmental growth and contributes to sustained psychological wellbeing.

## Evidence

### Schools as built environments

#### Macro-level assessment of how the overall physical environment directly impacts pupils’ lives

The built environment of schools has been empirically documented to impact pupils directly. For example, environmental psychologists demonstrate that the architectural environment of American primary schools predicts both attendance and academic achievement after controlling for other predictors such as socioeconomic status, ethnicity, school size, and teacher quality (Durán-Narucki, 2008). There is also a positive association between academic achievement and middle-school building conditions, mediated by social climate and student attendance (Maxwell, 2016). The destruction of school settings following natural hazard events prevents children from returning and receiving education (e.g., Mudavanhu, 2014; Adeagbo et al., 2016; Kousky, 2016). Thus, the maintenance of adequate school facilities is essential to protecting children’s right to education and their psychological wellbeing. These studies offer a macro-level assessment of how the overall physical environment directly impacts pupils’ lives, demonstrating that poorer facilities correlate with poor attendance and, therefore, poorer academic outcomes.

#### Micro-elements of spaces

Micro-elements of spaces that carry little meaning for adults have been shown to have great significance for young people (Koller and Farley, 2019). For example, Fleet and Britt (2011) found that children placed significance in a brick wall, which researchers initially saw as meaningless. Drawing on Stedman's (2003) ‘meaning-mediated model’, it is unlikely children were attached to the wall *per se*, but instead, the meaning represented by the wall, such as warmth and laughter (Koller and Farley, 2019). This is consistent with findings reported by Fleet and Britt (2011), that children often climbed and sat on the wall, creating new narratives of the wall’s significance through play. These person-place interactions also effectively subvert the adult narratives of safety and surveillance, which creates a sense of adventure and freedom that Scannell and Gifford (2016) theorize fosters positive attachments to place elements in children. Other studies have also documented differing affective responses to micro place elements. Koller and McLaren (2014) found that children shared spontaneous and charged emotional responses to hospitals’ physical design and decor that was not shared with adults. This demonstrates the importance of eliciting children’s insight into the meaning with which certain physical aspects of the school environment are endowed, as adults may be unaware of the meanings bestowed on seemingly mundane features of the school environment.

#### Place loss in disaster settings

The literature concerning place loss in disaster settings provides further insight into the relationship between bonds to physical places and psychological wellbeing. According to the disaster literature, the loss of physical place, most notably the home, is devastating for adults. Feelings of grief and emotional distress often accompany place loss, as cognitive-affective attachments are ruptured (Cox and Perry, 2011). This occurs beyond the initial disaster impact, as ongoing demolition leads to feelings of disorientation throughout the reconstruction period (Silver and Grek-Martin, 2015). Such disruption to one’s significant places can lead to “solastalgia,” which refers to the distress produced by environmental change (Albrecht et al., 2007). When “solastalgia” occurs, the environment no longer offers solace, sense of place and place identity, causing feelings of powerlessness that negatively impacts wellbeing (Warsini et al., 2014; Albrecht, 2019; Galway et al., 2019).

The impact of place loss on children is likely to become more salient for children post-disaster since they become aware of the attachment they had to the destroyed place and experience exacerbated feelings of distress due to the suddenness and unexpected nature of the impact (Relph, 1976; Cheng and Chou, 2015). To understand the symbolic significance of the loss of physical place, we turn to research that examines material loss through the lens of Moscovici's (2001) social representations theory. The home is often depicted as a place of safety, security and relaxation. Harries (2013) found that residents at risk of flooding in the UK are motivated to protect elements of the home that facilitate feelings of safety; the elements which function in this way are largely determined by social representations. However, traumatic or repeated damage to a home can threaten residents’ ontological security (Hawkins and Maurer, 2011; Harries, 2013), which occurs when a sense of trust in the stability of the home environment is undermined. Just as the home is associated with notions of continuity and safety for adults (see Mallett, 2004 for a review), many young people often see the school as a place of inclusion and safety (Butler et al., 2017). As children spend a large amount of time at school, a similar process may occur: the group-based sense of the school as a safe space (pre-disaster) may be challenged by a school becoming a place of danger, especially where there has been a threat to life (e.g., building collapse during an earthquake). This will have an emotional impact on the children. If both a sense of safety and of danger are held simultaneously, this may lead to what Moscovici (1984) terms ‘cognitive polyphasia’: representations may be plural and even contradictory, activated depending on the social context. For example, post-disaster children may simultaneously represent the school as a place of safety when among supportive peers and adults, and danger when witnessing infrastructural damage. Although this provides insight into the unique subjectivity of person-place bonds for children (versus adults) and peripherally informs our understanding of meanings made of schools, this area remains under-researched.

The process of rebuilding schools should be emphasized in community response and recovery plans. Scholars argue that involving children and youth in the design efforts is likely to benefit their wellbeing and cultivate positive place-attachment bonds to the place they have agency in creating. For example, Koller and Farley (2019) advocate for the right of children to be involved in the design of the spaces they inhabit. Research from disaster-affected areas has also shown that young people eager to be active in community recovery post-disaster (Peek, 2008; Taylor and Peace, 2015). Further, Pivik (2010) argues that children’s insights into place differ from adults’ and documents instances where children have identified barriers to the inclusion of disabled children that relevant adult stakeholders were unable to identify. The unique student perspective should be harnessed when physically rebuilding the school post-disaster to ensure the built environment meets young people’s needs, to truly ‘build back fairer’ (Sendai Framework for Disaster Risk Reduction 2015–2030) from a child-centered perspective.

### Schools as social arenas

This paper has thus far explored place meanings in terms of the physical, built environment. However, place is often considered a dual concept that incorporates a social element within the physical environment (Riger and Lavrakas, 1981; Scannell and Gifford, 2010; Koller and Farley, 2019). The relationship between both dimensions is symbiotic: bonds to physical places facilitate meaningful networks of relationships, just as meaningful relationships shape the meanings attributed to a physical place (Tuan, 1977; Hay, 1992). Schools are key sites for developing and maintaining social relationships, especially for children (Ellis, 2005). Yet, little research has explored or conceptualized the role of schools as social arenas and how they may come to exist as meaningful places for communities in disaster settings. This section outlines the social aspects of schools that serve significant functions in supporting children and youth in resilience building and psychosocial recovery.

#### Social support networks

For children in disaster settings, social support networks are vital resources. They introduce a plethora of protective psychosocial factors, such as sense of belonging and connection, into their lived experiences; these buttress psychological wellbeing. The notion that social support can ‘buffer’ the negative effects of stress on mental health for children and adults is well supported by contemporary research (Olstad et al., 2001; Cohen, 2004; Sharp et al., 2018; McGoron et al., 2020). As young people are happiest in places that facilitate access to peers and supportive adults, schools are especially significant social arenas for children in disaster settings because these environments provide access to multiple social actors (Chawla, 1992, cited by Ellis, 2005).

Schools provide opportunities for unique social connections that would not normally exist outside the school environment but are vital for safeguarding children’s wellbeing. For example, positive teacher-student relationships are protective against a series of risk factors for children, including depression, neglect, and bereavement (Wang et al., 2013; Sharp et al., 2018). These adult-child relationships are unique to the school settings (i.e., non-familial) and are often central in safeguarding children (Bhadra, 2016). After Hurricane Katrina, for young people required to change school, the positive support received from school staff was instrumental in supporting their wellbeing (Barrett et al., 2008). Teachers have been shown to go beyond their traditional roles to aid children in processing their disaster experiences, which involves regulating their own emotional responses to model effective coping (Mooney et al., 2021).

Schools also provide the greatest opportunity for friendships amongst children; these social relationships have been found to promote positive coping with psychological distress following a disaster. For example, a seminal study found children’s friendships to be the most salient providers of emotional support and coping assistance following a hurricane – more so than parents and teachers (Prinstein et al., 1996). Emerging insights from disaster settings also show friendships as drivers of returning to school. Empatika (2018) reports that young people in Palu, Indonesia, ranked highly their desire to return to school and reconnect with friends following the 2018 earthquake and tsunami. Play and sport are critical school-based activities that scaffold such peer-to-peer social connection; they foster psychological wellbeing and post-traumatic growth for children in disaster settings (Henley, 2005; Ray and Bratton, 2010; Goodyear-Brown, 2019). These activities are also drivers of childhood attachment to schools as play and sport are mechanisms through which children engage in valued person-place interactions and build a salient, positive place-bond with their schools (Scannell and Gifford, 2016).

After a disaster, the restoration and rebuilding of schools can symbolize the community’s resilience. School recovery allows children to return to normalcy and replace their emotional crises with the joy of being surrounded by other children and having a space to learn and play simultaneously (Fernandez et al., 2015; e.g., Telford and Cosgrave, 2006). Young people are more likely to engage in their usual activities (relative to local context) when they believe their teachers and friends support them, even when faced with considerable difficulties (Wickrama and Kaspar, 2007). It is especially important for children to re-engage in play, as it can alleviate traumatic stress (Fernandez et al., 2015). School recovery also benefits communities since it allows parents and guardians to focus on returning to their regular work, to sustain their families, while their children are at school. Returning to work activities also aids in the recovery of communities by contributing to economic recovery. Communities with high social capital and a history of community activities can take an active role in the process of economic recovery and contribute to its success and speed (Nakagawa and Shaw, 2004), which is essential in safeguarding children’s wellbeing.

#### Sense of connectedness between community members

Beyond what they symbolize for children, schools are important social arenas that facilitate and embed a vital sense of connectedness between community members. When a community harbors a sense of social connectedness (e.g., between family, friends, and neighbors) before a disaster, it benefits from a greater sense of community and camaraderie post-event, which then promotes its adaptive potential and resilient capacity (Thornley et al., 2014; Mutch, 2015). This effect is consistent with the ‘social cure’ in the context of public health, where group-based processes of social support and social integration are found to contribute to positive health outcomes (Haslam et al., 2018). However, communities require appropriate local infrastructure and a community hub for community connectedness to benefit preparedness, adaptation, and recovery in disaster settings (Thornley et al., 2014). Schools are well-documented as ideal community hubs for local disaster risk management planning: from a disaster risk perspective, schools are often built better and built safer; from a psychosocial perspective, they are familiar, stable, and often locally accessible environments in times of emergency (Leadbeater, 2013; Mutch, 2015; Oktari et al., 2018; Amini Hosseini and Izadkhah, 2020).

Overall, social networks play an important role in promoting wellbeing and resilience for children and communities. Cohesion in the community reduces the mental health burden on a community post-disaster. As social connections are documented protective factors, which buffer against (dis)stress, the importance of promoting and protecting social connection within communities in disaster settings is emphasized. Schools are meaningful places that act as repositories of social relationships with protective functions vital to safeguarding the wellbeing of children and their communities post-disaster.

### Schools as places of personal significance

The paper has thus far explored the meaningfulness of schools as built-environments and as social arenas, supporting the wellbeing and resilience of children in disaster settings. It moves on to the personal elements contributing to the person-place bond for these children. Specifically, we consider the nature of individual factors, such as lived experience, and group-based ties, such as religion and history, in disaster settings. We demonstrate how such psychosocial ties are often the cause and consequence of complex identity dynamics, which influence how schools are represented.

#### Place identity

The process of person-place bonding is marked by direct and indirect interactions between the person and the place (Scannell and Gifford, 2010). These interactions include what one does in the place (e.g., activities, social interactions) and how one feels about it (e.g., safe, comfortable, welcome). The paper has previously discussed the significance of what one does in the place, and now considers the significance of how one feels about a place.

When the meaningfulness of a place deepens over time, place attachments can evolve further into place identity: a process through which individuals come to incorporate cognitions about the physical environment into their self-definitions (Clayton, 2003; Gifford, 2014). Prolonged and repeated exposure imbue environments with meaning at the individual level, especially where these exposures provide opportunities for interaction with the environment and people within it (Lewicka, 2011; Anton and Lawrence, 2014). Repeated experiences of places in childhood contribute greatly to lifelong person-place bonds (Jack, 2010), which function in a similar way psychologically to an interpersonal attachment (e.g., Fried, 2000; Kelly and Hosking, 2008; Morgan, 2010; Donovan et al., 2012; Scannell and Gifford, 2014, 2017).

Child-school bonds can comprise strong affective, social, and cognitive elements that often endure throughout the life-course. The mechanisms that shape such feelings about a place and foster such enduring person-place bonds also shape sense of self and community, and influence psychological wellbeing (Ellis, 2005). There is a well-established link between identity consistency and psychological wellbeing in the academic literature (Rogers, 1951; Phinney et al., 2001; Greenaway et al., 2016, as cited in Suh, 2002); this includes place identity as it is a substructure of social identity, similar to gender or social class (Shamsuddin and Ujang, 2008; Qazimi, 2014). Though the complex relationship between identity and wellbeing is not clearly understood at present, the literature reflects that the gaining of identity and consistency can protect and enhance wellbeing (e.g., Ysseldyk et al., 2012; Praharso et al., 2017) while disruptions to identity can be severely problematic and are linked with deleterious outcomes.

When place identities are threatened, this can also lead to place-protective action, including local opposition to proposed developments to the built environment, such as wind turbines (e.g., Stedman, 2002; Devine-Wright, 2009). Following disasters, schools may be relocated, merged or closed if they are in a dangerous zone or no longer have enough pupils to remain viable. After the Canterbury earthquake, this was found to exacerbate the social and emotional stress of a trauma-affected community (Mutch, 2018). Although unexplored in the existing literature concerning schools, place identity may impact the community response to such closures, in the form of protests and legal action. Thus, identification with places that are stable, enduring environments can act as a protective mechanism, but can also become problematic and undermine resilience when left unmitigated. For example, Bihari et al. (2012) found that place attachment was associated with greater knowledge of wildfires and effective preparedness across six communities in the United States. However, Donovan et al. (2012) found place attachment to territory and landmarks interacted with culture to minimize evacuation behaviors for an Indonesian community under threat of volcanic activity. Beliefs in protective ceremonies and spiritual forces minimized evacuation behaviours.

### Meaning-making and appraising traumatic events

Schools may foster positive meaning-making outcomes because they exist as trusted places to which pupils and communities harbor positive cognitive and affective ties. As schools provide many resources to support their communities, accessing these places post-disaster can support adaptive coping practices. For example, being in a state of disrupted identity can be traumatic, especially for children who may not have the cognitive-affective tools to independently cope with or appraise events. Such adverse responses to traumatic experiences are due to the loss of ‘meaningful’ resources, including psychological and cultural resources (Hobfoll, 1989). Meaning-making is a key process through which people rectify disturbances in their sense of identity and maintain homeostasis (see Linley and Joseph, 2005; Park, 2010; King and Hicks, 2021). Finding meaning in a traumatic experience is an example of this process; which can be a stressful-inducing process, but also has the potential to lead to outcomes that enhance psychological wellbeing (King and Hicks, 2021). The meaning-making process is central to supporting recovery after disaster (Park and Blake, 2020). Schools are a vital resource drawn upon to influence how stressful a disaster is for its pupils. For example, after Hurricane Katrina, pupils who felt more connected to the school they had been relocated to reported fewer negative symptoms and more protective factors (Barrett et al., 2008). Schools may potentially act as places that can activate positive representations post-disaster as they are familiar spaces.

### Social representations and attachments to schools

To understand the psychosocial significance of schools in disaster settings, it is necessary to consider how they are valued by the communities within which they exist. The significance that individuals ascribe to a place is shaped by their lived experience in the context of the group-based meaning system of their community (Bruner, 1990; Van Patten and Williams, 2008); when a person’s social world has already identified a particular place as meaningful, the place symbolizes the group-based cognitions that formed the social representation(s) of that place, according to how or why it is valued by the group (Low and Altman, 1992; Joffe, 2003; Scannell and Gifford, 2010). In Social Representational terms, this section explores how the socio-cultural group representations of the school become internalized in the individual, that is, “how the ‘we’ becomes contained in the responses of the ‘I’” (Joffe, 2003, p.60).

Representations of schools influence how these places are encountered and utilized by their communities. Like religious institutions or museums, schools are associated with their specific function and are valued according to how these functions serve their communities. Social representations that circulate in a given culture refer to shared understandings of phenomena among a specific group (Joffe, 2003). While there is plurality of representations of school depending on socio-cultural, historical context, and group-specific ideologies, schools tend to be regarded as trustworthy places dedicated to the betterment of the character and knowledge of their pupils and cultivating their growth and resilience (Bryan, 2005; Luetz, 2019). In addition, in disaster settings, schools are also regarded as disaster risk reduction centers that support their communities at all stages of disaster: preparedness, response, and recovery (e.g., Sakurai et al., 2018). Schools facilitate the resilient recovery of post-disaster communities through educational disaster preparedness programs, staff safeguarding of children’s wellbeing peri- and post-event, and converting the building into a resource distribution center (Mutch, 2015). By bringing people together in a shared and familiar space, schools also promote a ‘culture of caring’ in communities post-disaster (Mutch, 2015). Each of these functions reinforces the community’s representation of their local school as a trustworthy place and further informs their representations of the significance of schools in disaster settings. As schools continue to support their communities in this way, shared beliefs that they are valuable becomes increasingly reinforced.

The significance of schools as symbols can also be understood by considering the psychosocial functions of memorials. Bonder (2009) explains that memorials help individuals and communities reappraise past traumatic events, while also existing to remind them about conduct and future events. It is well established that a sense of shared identity emerges amongst survivors in post-disaster settings, as the communal experience of the disaster prompts a sense of ‘we-ness’ (Rodríguez et al., 2006; Drury et al., 2016). While post-disaster gains in social capital are often short-term (Kaniasty and Norris, 1993), memorials can function to maintain these ties and evolve into a source of enduring community resilience (Ntontis et al., 2020). Memorials are also inextricably linked to space, thus if schools are used as a site of memorial the physical space of the school has the potential to become an ‘anchor of shared identity’ (Ntontis et al., 2020, p. 7) rooted in deeply person-centric elements of individual and group attachment. Therefore, schools can benefit from harnessing this sense of ‘we-ness’ experienced by shared fate after an earthquake in order to build resilient communities in highly seismic/disaster-prone regions.

## Conclusion

This paper has presented an evidence-based conceptualization of how and why schools exist as meaningful environments for children and their communities in disaster settings. It has considered the physical and social environments of schools and their significance at the individual and group levels (e.g., community). We have explicated a series of specific functions within each of these domains that make schools distinctly meaningful to their communities and highlighted the capacity of schools to foster community resilience and safeguard the wellbeing of children. We have also considered the nature of individual and shared ties to schools in disaster settings and demonstrated how such psychosocial ties are often the cause and consequence of complex identity dynamics that contribute to these environments being represented as symbolically significant. The mechanisms uncovered concerning how schools can provide these functions have important implications for the role of schools in mitigating the adverse impacts of disasters.

## Recommendations for future research

Based on the evidence concerning place bonds, we have attempted to synthesize existing frameworks to contribute a holistic conceptualization of how schools can bolster resilience in disaster settings. We intend for this knowledge to allow academics and practitioners in disaster preparedness and response to better understand and harness the school environments’ latent capacity to improve and protect community members before, during, and after disasters. For example, by exploring schools through a broad social psychological lens of place-bonds and attachment, we highlight that rebuilding the school’s physical infrastructure should be a priority in community disaster response and recovery efforts. We use a social representations theory approach to highlight that schools are community resources that can be used to foster community integration and cohesion, provide children a sense of stability and continuity, and provide pragmatic support to community members. Each of these functions safeguards wellbeing and fosters resilience across a series of psychosocial domains. Thus, while the loss of the physical place is traumatic, rebuilding a school after a disaster may symbolize community and communal continuity. Contemporary scholarly works have only begun to capture this notion; Dimension.ai, an opensource database that offers analytics of linked data, including grants, publications, datasets, patents, and policy documents, reports that interest in schools in disaster settings has been steadily increasing. This paper provides a foundation for dialogue to consider the socio-physical function of schools in communities and the lives of the individuals who spend time in these spaces (e.g., pupils, staff, parents/families). Future research should also aim to establish insight into how place attachment functions in seismic areas pre-disaster.

## Limitations

This paper expands on the contributions and syntheses provided by well-cited, prominent works concerning the person-place bond. However, it does not adhere to a single model or rigid conceptual framework. We acknowledge that this approach may have left some aspects of the place bond to schools unexplored and recommend that future research expand on our preliminary conceptual insights by empirically exploring the role and meaning of schools in disaster settings. We consider existing knowledge in social psychology, such as the person-place-process (Scannell and Gifford, 2010) and the self-other-environment (Gustafson, 2001) models of place attachment, but future research would benefit from approaching this topic through other lenses in order to deepen understanding. While this paper has regarded schools and the school environment as a place with positive valence, we also acknowledge that schools may not provide a positive experience for all people or in all places. For example, some schools have a more positive climate than others, which can impact mental health (Patalay et al., 2020). Just as social representations of the ‘home’ as a sanctuary do not reflect the lived experience of everyone (Mallett, 2004), we acknowledge that this may also be the case for representations of the school. Furthermore, some young people may have negative experiences of bullying, lack of belonging, and loneliness. This concern is especially important to note as children with special educational needs may be more vulnerable to feeling excluded (e.g., Cullinane, 2021). Future research should explicitly explore the critical role played by schools for children with special physical and educational needs, as schools may have different value and significance than captured in this paper. Finally, it must be recognized that the type of attachments pupils have may vary according to their age; future research should empirically explore the psychosocial role of schools for children and adolescents separately.

## Author contributions

E-MP, EP, and HJ: conceptualisation. E-MP and EP: writing – original draft preparation. E-MP, EP, RO, and HJ: writing – review and editing. HJ: supervision and funding acquisition. All authors have read and agreed to a published version of the manuscript.

## Funding

This research was funded by the UK Research and Innovation (UKRI)/Economic and Social Research Council (ESRC) Global Challenges Research Fund (GCRF) for Equitable Resilience [grant number ES/T002956/1].

## Conflict of interest

The authors declare that the research was conducted in the absence of any commercial or financial relationships that could be construed as a potential conflict of interest.

## Publisher’s note

All claims expressed in this article are solely those of the authors and do not necessarily represent those of their affiliated organizations, or those of the publisher, the editors and the reviewers. Any product that may be evaluated in this article, or claim that may be made by its manufacturer, is not guaranteed or endorsed by the publisher.
